# On-the-Scene Hyaluronan and Syndecan-1 Serum Concentrations and Outcome after Cardiac Arrest and Resuscitation

**DOI:** 10.1155/2019/8071619

**Published:** 2019-04-17

**Authors:** V. Bogner-Flatz, M. Braunstein, L. E. Ocker, T. Kusmenkov, J. Tschoep, L. Ney, W. Böcker, T. Annecke

**Affiliations:** ^1^Department of Trauma Surgery, Ludwig Maximilian University Hospital Munich, Nussbaumstr. 20, 80336 Munich, Germany; ^2^Department of Anaesthesiology, Ludwig Maximilian University Hospital Munich, Nussbaumstr. 20, 80336 Munich, Germany; ^3^Department of Anaesthesiology and Intensive Care Medicine, University Hospital of Cologne, Cologne, Germany

## Abstract

**Background:**

It is not predictable which patients will develop a severe inflammatory response after successful cardiopulmonary resuscitation (CPR), also known as “postcardiac arrest syndrome.” This pathology affects only a subgroup of cardiac arrest victims. Whole body ischemia/reperfusion and prolonged shock states after return of spontaneous circulation (ROSC) may both contribute to this devastating condition. The vascular endothelium with its glycocalyx is especially susceptible to initial ischemic damage and may play a detrimental role in the initiation of postischemic inflammatory reactions. It is not known to date if an immediate early damage to the endothelial glycocalyx, detected by on-the-scene blood sampling and measurement of soluble components (hyaluronan and syndecan-1), precedes and predicts multiple organ failure (MOF) and survival after ROSC.

**Methods:**

15 patients after prehospital resuscitation were included in the study. Serum samples were collected on the scene immediately after ROSC and after 6 h, 12 h, 24 h, and 48 h. Hyaluronan and syndecan-1 were measured by ELISA. We associated the development of multiple organ failure and 30-day survival rates with these serum markers of early glycocalyx damage.

**Results:**

Immediate serum hyaluronan concentrations show significant differences depending on 30-day survival. Further, the hyaluronan level is significantly higher in patients developing MOF during the initial and intermediate resuscitation period. Also, the syndecan-1 levels are significantly different according to MOF occurrence.

**Conclusion:**

Serum markers of glycocalyx shedding taken immediately on the scene after ROSC can predict the occurrence of multiple organ failure and adverse clinical outcome in patients after cardiac arrest.

## 1. Introduction

Sudden cardiac arrest is one of the leading causes of death worldwide. Approximately 822,000 deaths per year are related to this condition in Europe and in the United States of America [1, 2]. Despite extent resuscitation strategies, morbidity and mortality after cardiac arrest are continuously high [3]. 70% of patients with initial ROSC will not survive the postresuscitation period [4]. A majority of these patients develop systemic inflammation that frequently entails complex pathophysiological condition with extensive multiple organ injury [5, 6]. This is subsumed as postresuscitation syndrome [7, 8]. The latter has been described as to be very similar to sepsis and sepsis-like syndromes. The syndrome can be initiated either by the initial ischemia/reperfusion injury during cardiac arrest and subsequent resuscitation or by a prolonged shock state as a result of persistent cardiogenic dysfunction after initial successful resuscitation [9]. Overall, systemic inflammation with consecutive macro- and microcirculatory dysfunction further aggravates organ dysfunction mediated as well by direct leucocyte transmitted by leucocyte-independent tissue damage [6]. Geppert et al. postulate the presence of a “systemic inflammatory response syndrome” (SIRS) in 66% of their patients after cardiac arrest and ROSC [8].

Though their plenitude is well recognized, the exact pathophysiological mechanisms of such complex syndromes like ischemia/reperfusion and subsequent systemic inflammation are still not completely understood. As supported by actual scientific findings, endothelial dysfunction and damage to the endothelial glycocalyx may play a significant role in ischemia/reperfusion injury and initiation of early SIRS. Endothelial glycocalyx regulates and alternates several mechanisms under physiological and pathological conditions like leucocyte-endothelial interaction, vascular barrier function, and transmission of flow-mediated shear stress [10]. Both ischemia and hypoxia are capable to damage the endothelial glycocalyx in the experimental models [11–13]. Rehm et al. reported the elevated syndecan-1 and hyaluronan plasma levels as markers of glycocalyx shedding after local and regional ischemia-reperfusion in patients who underwent vascular and cardiac surgery [14]. Soluble parts of the endothelial glycocalyx can be detected in plasma of patients during septic shock [15]. The extent of glycocalyx shedding and thereby rising plasma levels of its components strongly correlate with a higher rate of morbidity and mortality, probably triggering independently critical illness after trauma and ischemia [16]. In addition, inflammatory mediators like TNF-alpha induce a modification of the endothelial glycocalyx [11, 17]. Grundmann et al. investigated the endothelial damage markers in patients after cardiac arrest and found the raised levels of syndecan-1, heparan sulfate, and hyaluronan in negative correlation to survival [3]. However, they choose a six-hour time frame for the first blood analysis in their study. Bro-Jeppesen et al. reported a positive correlation of IL-6 as an inflammatory marker and syndecan-1 serum levels with the extent of postresuscitation syndrome but not with the outcome [18].

The aim of our study was to investigate the role of glycocalyx shedding immediately after ROSC following out-of-hospital cardiac arrest. We were especially interested in a potential correlation of the early post-ROSC concentration of the glycocalyx components hyaluronan and syndecan-1 as biomarkers for the development of postcardiac arrest syndrome and multiple organ failure.

## 2. Materials and Methods

### 2.1. Study Design and Population

The study was designed as a prospective, multicentre observational trial. It was approved by the Institutional Ethical Review Board at Ludwig Maximilian University, Munich, Germany (decisions: 282/01), and written informed consent was obtained from each patient's next of kin and/or from patients regaining consciousness after cardiac arrest. The study was performed in accordance with the Declaration of Helsinki of the World Medical Association and its amendments and European Union guidelines for good clinical practice.

Patients (≥18 years) who were successfully resuscitated from out-of-hospital cardiac arrest (OHCA) with sustained unconsciousness (GCS < 8) after ROSC between January 2003 and March 2012 were enrolled in this prospective study. Exclusion criteria were terminal disease, acute infectious disease, immunosuppressive therapy, major trauma, pregnancy, severe refractory cardiogenic shock at time of admission, and treatment with the intra-aortic balloon pump or left ventricular assist device.

Survivors of OHCA were admitted to one of the four participating maximum care hospitals in the city of Munich after successful resuscitation. Therapy and laboratory testing were standardized in all participating centres. They received intensive care treatment according to current resuscitation guidelines including mechanical ventilation, fluid substitution, antibiotic therapy, SIRS/sepsis management, and targeted temperature management. Patients were sedated and received adequate analgesia according to the standard of each ICU. Percutaneous coronary intervention was performed if necessary.

### 2.2. Data Collection

Prehospital data regarding cardiac arrest, initial heart rhythm, witnessed arrest, time to initiation of CPR, administration of bystander CPR, and time to ROSC were systematically collected according to Utstein guidelines. Additionally, the following variables were recorded for each patient: demographic data, clinical parameters and comorbidities, presence of cardiogenic shock, renal replacement therapy, and duration of stay in the intensive care unit and in hospital.

### 2.3. Blood Sampling

Blood samples were drawn immediately after ROSC by the attending emergency physician and subsequently 6, 12, 24, and 48 hours after ROSC. Serum samples were stored immediately at -80°C until further analysis.

Hyaluronan concentrations were quantified by enzyme-linked immunosorbent assay (Hyaluronan Enzyme-Linked Immunosorbent Assay Kit (HA-ELISA) K-1200; Echelon Biosciences Inc., Salt Lake City, UT, USA). Syndecan-1 concentrations were quantified by sandwich enzyme-linked immunosorbent assay Human sCD138 (Syndecan-1) ELISA Kit (Diaclone SAS 50.640; Diaclone SAS, Besancon Cedex, France).

### 2.4. Data Analysis

This study was designed to (1) analyse the serum levels and time course of syndecan-1 and hyaluronan immediately after ROSC and within the first 48 h after successful cardiopulmonary resuscitation and (2) associate serum concentrations with distinct clinical outcome parameters. Patients were retrospectively distributed to subgroups in regard to the development of multiple organ failure and 30-day mortality. Multiple organ failure (MOF) was defined using a MOF score as described by Goris et al. and modified by Lefering et al. Patients with a MOF score equal or greater than the median MOF score in the entire study population (which turned out as 6 points, see Results) were indexed as multiple organ failure “high.” Survival was defined as 30-day survival after cardiac arrest and return of spontaneous circulation.

Statistical analyses were performed using the SPSS Statistics Version 22.0 (IBM Corporation; Armonk, NY, USA). To take sequential measurements into account and to evaluate target dynamics in particular, we selected a statistical model for repeated measure analyses (repeated measures MANOVA, SPSS general linear model). The following parameters were included in the model: time to indicate significant protein concentration changes, development of multiple organ failure, and 30-day survival. Consequently, we could detect dynamic changes and identify the influence of MOF and/or survival on the type and extent of protein concentration changes. The level of significance was set to 0.05. If findings were statistically significant, the single instance of time was post hoc evaluated using the nonparametric Mann-Whitney*U*test. If complete clinical data sets were not available, patients were excluded from subsequent analysis.

## 3. Results

### 3.1. Clinical Baseline Characteristics

Fifteen patients fulfilled inclusion criteria and were enrolled in this study from January 2003 to March 2012. Mean age was 61.7 years. 20% of all patients were female. MOF score was calculated in 13 patients (87%). Median MOF score was 6 with a minimal value of 4 points and a maximal value of 8 points. Multiple organ failure developed in four patients (31%; MOF < 6: *n* = 9; MOF ≥ 6: *n* = 4). 60% survived after cardiac arrest and return of spontaneous circulation (30-day survival; survivors *n* = 9; nonsurvivors *n* = 6). Survivors were 60 years by mean; mean age of nonsurvivors was 64.3 years. Mean MOF score was 6.67 points in nonsurvivors. Survivors showed a mean MOF score of 5.29 points. Detailed clinical data is listed in Table 1.

### 3.2. Serum Concentrations

**Figure 1:**
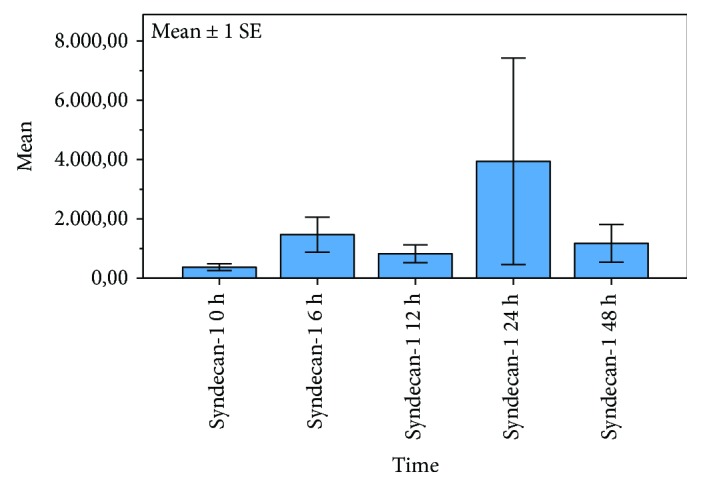
Serum levels of syndecan-1 within 48 h after out-of-hospital cardiac arrest. Undulant serum levels of syndecan-1 without significant dynamics after resuscitation; bars represent mean values with error bars representing ±1 SE. MANOVA analysis for repeated measurements; *p* = 0.907.

**Figure 2:**
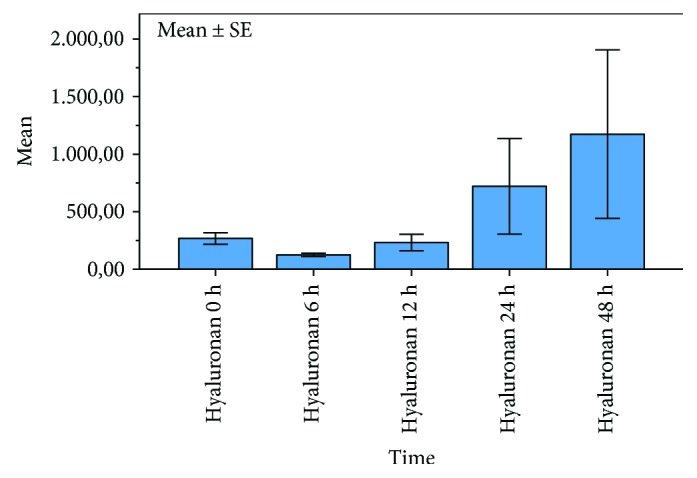
Serum levels of hyaluronan within 48 h after out-of-hospital cardiac arrest. Significant increase of hyaluronan levels within 48 h after resuscitation; bars represent mean values with error bars representing ±1 SE. MANOVA analysis for repeated measurements; *p* = 0.043.

The dynamic of hyaluronan and syndecan-1 concentrations within the first 48 h after cardiac arrest is illustrated in Figures 1 and 2. Syndecan-1 levels (Figure 1) showed the undulant values without significant changes within 48 h after cardiac arrest. Hyaluronan levels (Figure 2) showed a significant increase within 48 h after ROSC.

### 3.3. Serum Concentrations and Outcome (30-Day Survival)

#### 3.3.1. Hyaluronan and Survival

**Figure 3:**
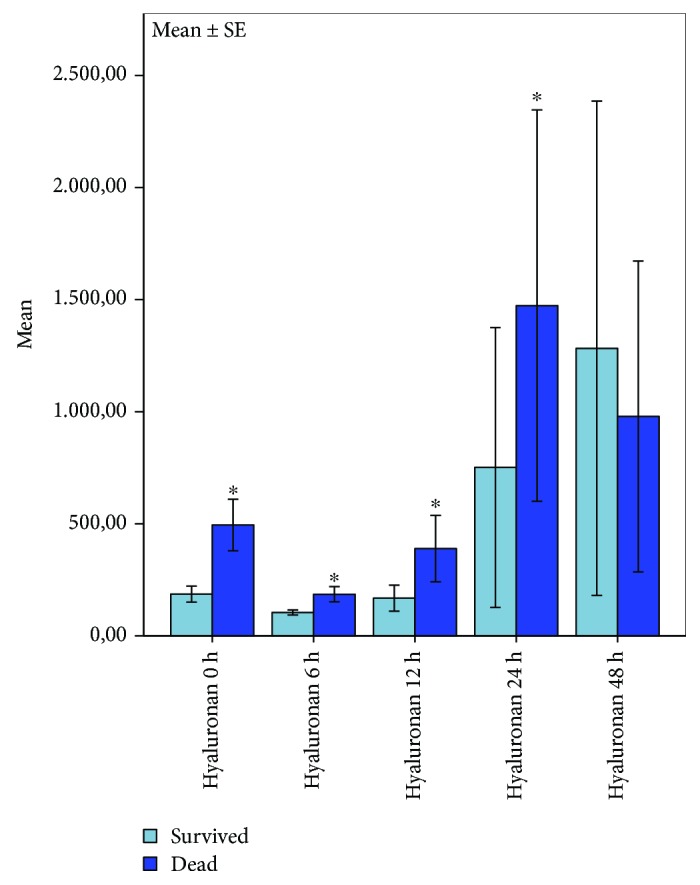
Plots of correlation between hyaluronan levels and 30-day survival 0 h-48 h after cardiac arrest. Significant correlation of hyaluronan levels and survival 0 h-24 h after ROSC; bars represent mean values with error bars representing ±1 SE. MANOVA analysis of variances and the post hoc ^∗^Mann-Whitney *U* test; *p* ≤ 0.05; survivors *n* = 9, nonsurvivors *n* = 6.

Our results showed a significant association between hyaluronan concentrations, time, and 30-day survival detected by multivariate testing of variances (Figure 3). Post hoc analysis revealed a significantly higher immediate early concentration of hyaluronan in nonsurvivors when compared to survivors on the scene after ROSC (0 h: *p* = 0.012) and in the following 24 hours after ROSC (6 h: *p* = 0.018, 12 h: *p* = 0.036, and 24 h: *p* = 0.036). There was no statistically significant difference 48 h after ROSC (*p* = 0.438).

#### 3.3.2. Hyaluronan and Multiple Organ Failure

**Figure 4:**
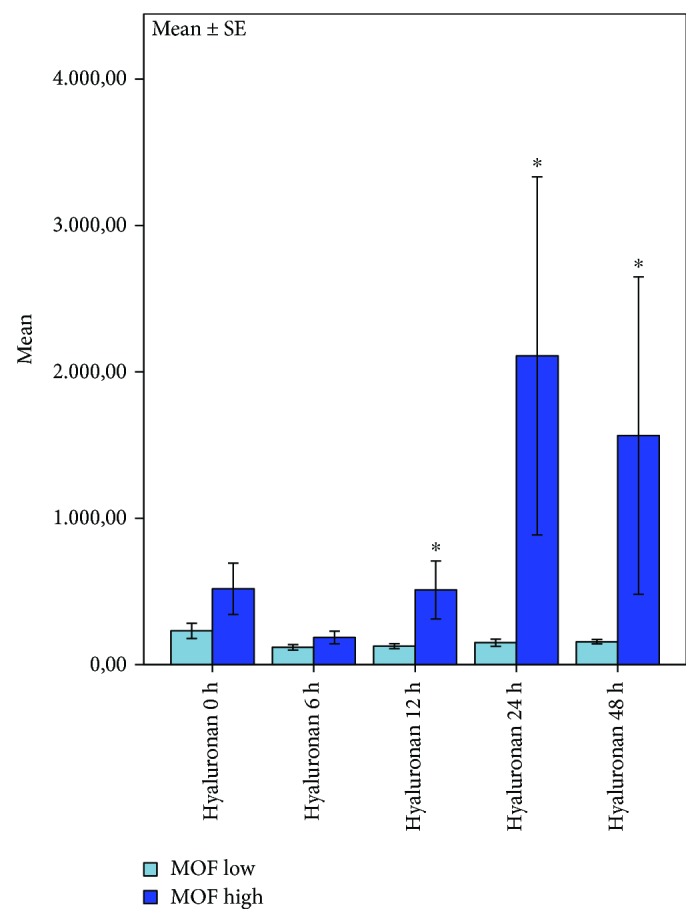
Plots of correlation between hyaluronan concentrations and MOF score 48 h after cardiac arrest. Significant correlation between hyaluronan release, time, and multiple organ failure 0 h-48 h after ROSC; bars represent mean values with error bars representing ±1 SE. ANOVA analysis of variances and the post hoc Mann-Whitney rank sum test; *p* ≤ 0.05; MOF ≤ 6: *n* = 9, MOF > 6: *n* = 4.

Patients who developed a multiple organ failure showed higher hyaluronan serum levels when compared to patients with MOF scores less than 6 points (Figure 4). Our results state a significant association between hyaluronan concentrations, time, and development of multiple organ failure after ROSC (*p* = 0.035) measured by multivariate analysis of variances. Patients developing MOF show significant higher hyaluronan concentrations at 12 h after ROSC (12 h: *p* < 0.011, 24 h: *p* < 0.06, and 48 h *p* < 0.09; Mann-Whitney *U* test).

#### 3.3.3. Syndecan-1 and Survival

Syndecan-1 serum levels were higher in nonsurvivors 0 h-12 h after cardiac arrest. 24 h and 48 h after cardiac arrest, syndecan-1 release was higher in survivors. Syndecan-1 serum levels were not significantly associated with survival after ROSC (not shown).

#### 3.3.4. Syndecan-1 and Multiple Organ Failure

**Figure 5:**
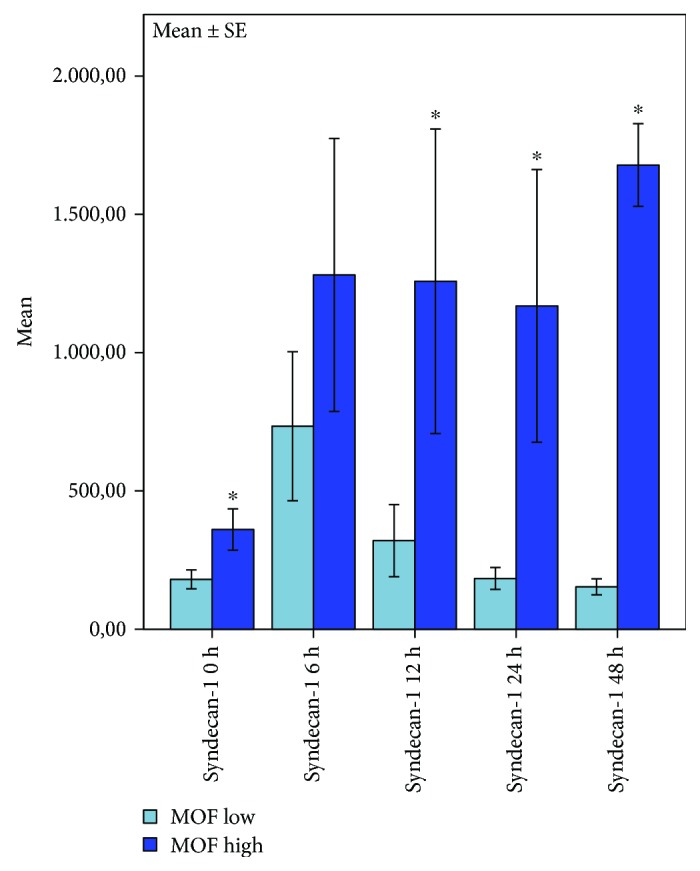
Plots of correlation between syndecan-1 levels and MOF score 48 h after cardiac arrest. Highly significant correlation of syndecan-1, time, and multiple organ failure (MOF > 6); bars represent mean values with error bars representing ±1 SE. ANOVA analysis of variances and the post hoc Mann-Whitney rank sum test; *p* ≤ 0.05; MOF ≤ 6: *n* = 9, MOF > 6: *n* = 4.

Syndecan-1 serum levels were higher in patients suffering from multiple organ failure 0 h-48 h after cardiac arrest. Interestingly, these differences already can be observed immediately after ROSC and remain the entire observation period. Multivariate analysis of variances testing syndecan-1 levels, time, and development of multiple organ failure showed a highly significant correlation presenting higher syndecan-1 levels in patients suffering from multiple organ failure (MOF > 6) within 48 h after ROSC (*p* = 0.001) (Figure 5).

## 4. Discussion

To the best of our knowledge, this is the first study analysing glycocalyx shedding in blood samples taken immediately after resuscitation in out-of-hospital cardiac arrest. Up to now, the first sample obtained immediately after ROSC allows conclusions about the initial total body ischemia/reperfusion injury induced by cardiac arrest and resuscitation, independent from the development or persistence of subsequent shock states which can influence the results in subsequent taken blood samples. Hyaluronan is continuously increasing with an initial peak at 0 h and further increasing levels starting as early as 12 h after ROSC. In contrast, syndecan-1 levels start initially lower and only show an undulant concentration curve over time. In particular, the first finding postulates that endothelial activation and damage to the glycocalyx appear to be a very early effect of cardiac arrest and resuscitation. Only few clinical studies address data comparable to ours. Grundmann et al. found both glycocalyx parts in the patient serum after cardiac arrest and ROSC over a time course of 72 h. However, the first blood sample was collected in a 6 h time frame after the event, likely to be influenced by subsequent shock states and intensive care therapy [3]. Bro-Jeppesen et al. reported elevated concentrations for syndecan-1 on admission with a drop at 24 h [18]. Both findings are in line with our 6 h time point. However, we noted that syndecan-1 shows a lower concertation at the 0 h time point as compared to hyaluronan. Segregated and free circulating syndecan-1 is known to be degraded quickly [19]. Therefore, our study and the two others mentioned perfectly complement one another but have limitations in direct comparability.

Ischemia-induced glycocalyx damage develops early and is a strong trigger for local and systemic inflammatory mediator release [20–22]. Bro-Jeppesen et al. found a significant correlation between syndecan-1 and IL6 [18]. IL-6 has often been suggested as a suitable inflammatory and outcome marker for postresuscitation syndrome [20]. After a peak at 24 hours, IL-6 drops again but the extent of systemic inflammation is further ongoing. Peberdy et al. hypothesized that proinflammatory cytokines may initiate but not perpetuate the postresuscitation syndrome [23]. In contrast to IL6, we found that syndecan-1 is still being released at 24 h, hypothetically due to other factors than the initial cardiac arrest-induced ischemic insult, like further persistent shock states or aggressive vasopressor and fluid treatment. Therapeutic interventions do also significantly influence endothelial integrity. For example, vasopressor and inotropic medication for therapeutic use are known to cause endothelial damage [24] whereas some other interventions like steroids and volatile anaesthetics seem to have rather protective effects [12, 25]. Most patients after resuscitation—as seen in our study collective—need some sort of circulatory support during intensive care treatment, which is inescapable on one side but might also be a trigger for complications.

This fits to our findings that patients with a greater extent of multiple organ failure—calculated by MOF score—consistently exhibited higher levels of hyaluronan and syndecan-1. Syndecan-1 already differentiates MOF extremely early after ROSC and may thereby be useful as an early indicator for subsequent development of the postresuscitation syndrome. This needs further evaluations in a study with a larger patient collective. Furthermore, we could show a significant association between the hyaluronan levels and the unfavourable outcome. Therefore, our study could show an association between endothelial shedding and postresuscitation syndrome, consecutive multiple organ failure, and finally the adverse outcome. In this context, the extent of ischemia-induced endothelial damage seems to be a detrimental factor for the further clinical course of this patient collective [6, 10, 22]. Similar effects have been described for other severe systemic inflammatory syndromes like sepsis or major burn injury and major trauma [26, 27]. Frydland et al. reported the raised syndecan-1 levels in patients after STEMI and cardiogenic shock [28]. This condition may also play a role in some of our patients; unfortunately, we have not been able to separate our patients according to the initial cause of cardiac arrest for statistical reasons.

The very early patient access and first blood sampling in this delicate patient collective entail relevant logistics and differentiate our investigation from several other studies with a similar patients collective [3, 18, 23, 29].

Patients with relevant comorbidities like severe chronic illness, acute or chronic infections, pharmaceutical immunosuppression, or malignancies were not included to reduce influence of confounding factors. In an investigation on human blood, these confounding factors were shown to have profound influence on endothelial and glycocalyx damage and systemic inflammation [10, 13, 15, 30]. These circumstances lead to a relatively small patient collective in our investigation.

## 5. Limitations

Main limitation of our work is the small sample size which is due to the very challenging preclinical and clinical setting. Our emergency physicians collected the first blood sample on the scene immediately after ROSC in order to evaluate serum protein level changes as early as possible. Afterwards, patients were admitted to four different hospitals in town, which entailed challenging logistic efforts. Though there is an immense effort in improving the management of OHCA, survival rates are still quite poor. Patients were only included in the study if the first blood sample on the scene was feasible and if the patient could be admitted to hospital. These entry criteria lead to a restricted patient size in our study and to a certain bias in survival rates as compared to overall survival rates studying the outcome in OHCA, for example, in the PARAMEDIC-2 trial [31]. Due to the association found in our investigation, we plan a larger prospective trial, which will allow for further differentiations.

## 6. Conclusion

Postresuscitation multiple organ failure is still one of the major causes of mortality and morbidity in patients with return of spontaneous circulation after cardiac arrest. Ischemia/reperfusion injury, persisting shock states, and systemic inflammation lead to the complex postresuscitation syndrome. In this context, damage to the glycocalyx seems to be a major trigger for the maintenance of the latter. We present for the first time the patient serum levels of free circulating hyaluronan and syndecan-1 immediately after ROSC, complemented by serial measurements in the postresuscitation period for 48 h. Our data support that the extent of glycocalyx shedding shows a characteristic time course and is significantly associated with multiple organ failure and adverse outcome. In line with the actual literature, we thereby postulate a strong role of endothelial damage expressed by glycocalyx shedding in the pathophysiology of postresuscitation disease. Our investigation is limited by a limited number of enrolled patients, owing to the study design, sumptuous logistics, and targeted patient selection, but it is though promising and encouraging for further research.

## Figures and Tables

**Table 1:** Detailed clinical data (*n* = 15).

No.	Age	Sex	Time of death	MOF high	Witnessed arrest	Initial heart rhythm	Cause of CA
1	56	M		N	Y	VF	MI
2	45	M		N	N	VF	MI
3	70	M	3 d	N	N	VF	MI
4	62	M		N	N	VF	MI
5	72	F		N	N	A	LE
6	54	M	8 d	Y	Y	VF	Unknown
7	61	M		Y	Y	VF	MI
8	90	M		N	Y	VF	Arrhythmia
9	51	M		N	N	VF	MI
10	62	F	7 d	N	N	VF	MI
11	69	F		N	Y	VF	MI
12	72	M	11 d	Y	Y	A	MI
13	45	M	2 d	Y	N	VF	MI
14	83	M	4 d	N	N	VF	MI
15	34	M		N	Y	A	LE

A: asystole; d: day; LE: lung embolism; F: female; M: male; MI: myocardial infarction; N: no; VF: ventricular fibrillation; Y: yes.

## Data Availability

The data used to support the findings of this study are available from the corresponding author upon request.
